# The chloroplast genome of *Archontophoenix alexandrae* (Arecaceae): an important landscape tree for the subtropics

**DOI:** 10.1080/23802359.2020.1715290

**Published:** 2020-01-20

**Authors:** Ying-ying Liu, Zhan-jiang Zhang, Shao-feng Jiang, Dong-lan Wang, Ling-jian Gui

**Affiliations:** a Guangxi Botanical Garden of Medicinal Plants, Nanning, Guangxi, China;; b Guangxi Key Laboratory of Tumor Immunology and Microenvironmental Regulation, Guilin Medical University, Guilin, Guangxi, China

**Keywords:** Arecaceae, landscape tree, chloroplast genome, phylogenetic analysis

## Abstract

*Archontophoenix alexandrae,* known as king palm, is an important landscape tree for the subtropics and potential sources of dietary fiber. In this study, the complete chloroplast genome of *A. alexandrae* was determined through Illumina sequencing method. The chloroplast genome was 159,196 bp in length and contained a small single-copy region (17,763 bp), a large single-copy region (87,055 bp) and a pair of IR regions (27,189 bp). 135 genes were determined in the *A. alexandrae* chloroplast genome, including 86 CDS, 39 tRNA genes, and 8 rRNA genes. *Archontophoenix alexandrae* showed the closest relationship with *Veitchia arecina* in the phylogenetic analysis.

*Archontophoenix alexandrae* (F. Muell.) H. Wendl. et Drude (Arecaceae) is an arbor-like plant endemic to the east of Australia (Dowe and Hodel 1994), known as king palm. The tree can reach 25 m, and is widely cultivated in the gardens of subtropical areas, such as Yunnan, Hainan and Hong Kong (Jim 1997), which is a characteristic tree for landscaping in tropical and subtropical areas. The king palm flours contain high contents of total dietary fiber and total ash (Vieira NL et al. 2010), which has the potential to replace part of wheat flour, rice flour and corn starch in food making (Vieira MA et al. 2008; De Simas et al. 2009). Here, we assembled and analyzed the complete chloroplast genome of *A. alexandrae* (GenBank accession number MN812494) based on the next-generation sequencing method.

The healthy leaves of *A. alexandrae* were collected from Guangxi Botanical Garden of Medicinal Plants (22°51′23″N, 108°22′5″E). The voucher specimens (lyy01) were preserved in the herbarium of Guangxi Botanical Garden of Medicinal Plants (GXMG). The whole genome sequencing was sequenced using Illumina genome platform (HiseqPE150). The clean data were assembled via NOVOPlasty (Dierckxsens et al. 2017) to complete chloroplast genome. The assembled chloroplast genome was annotated using PGA-master (Qu et al. 2019) with the chloroplast genome annotations of related species and *Amborella trichopoda* (GenBank number AJ506156) as references, and corrected manually in Geneious 10.2 (Kearse et al. 2012). 11 other complete chloroplast genomes of Arecaceae were achieved from the National Center for Biotechnology Information (NCBI). The data set was aligned using MAFFT (Katoh et al. 2002). A maximum likelihood (ML) tree was constructed by Mega 7 (Kumar et al. 2016) with 1000 bootstrap.

The chloroplast genome of *A. alexandrae* shows a four-segment structure with the length of 159,196 bp, containing a small single-copy region (17,763 bp), a large single-copy region (87,055 bp) and a pair of IR regions (27,189 bp). 135 genes were determined in the chloroplast genome, including 86 CDS, 39 tRNA genes and 8 rRNA genes. The total GC content of complete chloroplast genome was 37.2%.

Phylogenetic analysis shows that *A. alexandrae* has the closest genetic relationship with *Veitchia arecina* (Figure 1). *Areca vestiaria* also has a close relationship with *Archontophoenix alexandrae*. The complete chloroplast genome will provide useful information for biological and protection research for *A. alexandrae*.

**Figure 1.**
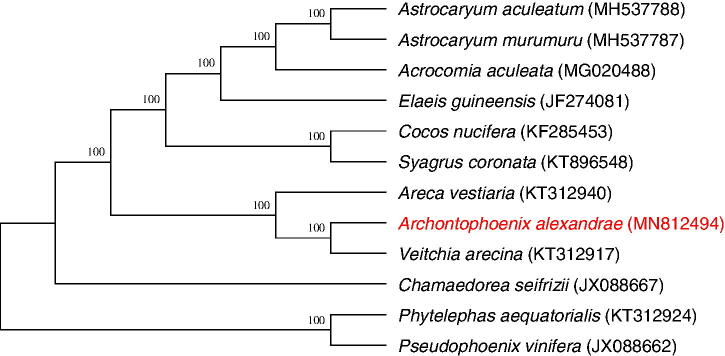
Phylogenetic tree reconstruction of 12 taxa of Arecaceae based on the complete chloroplast genome sequences. Numbers above the branches are the bootstrap values of ML.
